# Subjective Family Socioeconomic Status and Peer Relationships: Mediating Roles of Self-Esteem and Perceived Stress

**DOI:** 10.3389/fpsyt.2021.634976

**Published:** 2021-03-24

**Authors:** Xia Bai, Liping Jiang, Qi Zhang, Ting Wu, Song Wang, Xiaoying Zeng, Yanjia Li, Li Zhang, Jingguang Li, Yajun Zhao, Jing Dai

**Affiliations:** ^1^The Clinical Hospital of Chengdu Brain Science Institute, MOE Key Lab for Neuroinformation, University of Electronic Science and Technology of China, Chengdu, China; ^2^Chengdu Mental Health Center, The Fourth People's Hospital of Chengdu, Chengdu, China; ^3^Department of Radiology, Huaxi MR Research Center, West China Hospital of Sichuan University, Chengdu, China; ^4^School of Nursing, Chengdu Medical College, Chengdu, China; ^5^College of Teacher Education, Dali University, Dali, China; ^6^School of Education and Psychology, Southwest Minzu University, Chengdu, China

**Keywords:** subjective family social-economic status, peer relationships, self-esteem, perceived stress, Chinese adolescents

## Abstract

This study explored the relationships between subjective family socioeconomic status (FSES), self-esteem, perceived stress, and perceived peer relationships among Chinese adolescents. A total of 1,353 adolescents (age range: 15–19 years) were asked to complete a questionnaire. Mediation analysis revealed that subjective FSES influenced perceived peer relationships in three ways: first, through the mediating effect of perceived stress; second, through the mediating effect of self-esteem; and third, through the serial mediating effects of perceived stress and self-esteem. The results remained significant after controlling for parental education. In addition, a contrast analysis showed no significant differences in the mediating effects of self-esteem and perceived stress. Thus, we suggest that steps should be taken to improve adolescents' self-esteem and reduce their stress through training interventions and preventive measures, to help them improve their perceived peer relationships and reduce adverse effects associated with low subjective FSES.

## Introduction

Scholars have long considered peer relationships to be among the most important characteristics of adolescence. The transition from childhood to adolescence leads to changes in an individual's social environment and social norms, making peer relationships more prominent during this developmental period (1). According to group socialization theory, peers have greater influence on adolescent development than parents (2). Adolescents may spend more time with their peers, while adult supervision tends to be reduced, and they place more value on peer expectations and opinions (3).

Peer relationships are interpersonal relationships established and developed through the process of communication between individuals at similar levels of psychological development (3). Perceiving good peer relationships can help adolescents gain a sense of belonging within their peer group (4), and are critical to the positive development of their cognitive and social skills, as well as academic adaptation (5). In contrast, perceiving negative peer relationships make it difficult for adolescents to control their emotions (6), which may even lead to psychological problems such as anxiety, depression, and other mental disorders (7). It is therefore necessary to explore the factors that influence peer relationships.

Although peer relationships are an important part of adolescent development, not all adolescents have the ability to build beneficial peer relationships (8). Some research has indicated that family socioeconomic status (FSES) plays a role in peer relationships and its influence cannot be ignored (9). For example, a study of teenagers in 35 countries demonstrated that boys and girls from a lower-socioeconomic status group had fewer close friends and poorer peer relationships (10). Furthermore, adolescents with low FSES are often considered by their peers to have low ability and social status, which, in turn, leads them to experience exclusion thereby increasing their risk of developing adverse peer relationships (8, 11).

These previous studies indicated that FSES may be an important factor affecting peer relationships; however, there are two categories of FSES: one measured objectively by income and other observable factors, and the other measured subjectively by individuals' self-reported relative status among peers (12). Compared with objective measures, subjective FSES reflects a cognitive assessment of a person's relative social status and captures its subtle aspects more effectively (13, 14). A meta-analysis including 142,836 participants from 38 independent studies found that subjective SES was more significantly associated with physical and mental health (15). Therefore, we focused on subjective FSES to explore its association with peer relationships among Chinese adolescents.

It is well-known that personal development must be understood through the interplay of environment and individual characteristics, such as Bronfenbrenner's bioecological model of development (16) and family systems theory (17). Family provides the earliest environment for children's socialization and plays a fundamental role in child psychology (18). It not only directly influences an adolescent's internalized and externalized behavior problems, but also indirectly influences behavior problems through the individual's ego system (19). Self-esteem is one of the core components of the ego system, which is not only affected by family factors, but also affects the emotional health of adolescents (20). Studies have found that children who face financial difficulties at home during adolescence have lower self-esteem, higher levels of distress, more social and emotional problems, and are more sensitive and negative in their relationships with others (21, 22). In addition to the effect of family environment on self-esteem, levels of perceived stress have also been shown to vary widely among families (23). The Family Stress Model (FSM) suggests that economic stress can continue to affect adolescent development by influencing parents' negative emotions (24). Adolescents with financial difficulties are more likely to be exposed to negative family relationships and to be more sensitive to future stressors (25), and putting them at greater risk of negative emotions and developmental outcomes, as well as problems such as hostility and maladjustment (26–28). Consequently, in the present study we also explored the underlying mechanism in the relationship between subjective FSES and peer relationships from the perspective of psychological characteristics (i.e., self-esteem) and cognitive evaluation of one's external environment [i.e., perceived stress (29)].

Self-esteem was once considered the most important personality variable for understanding human behavior (30), and while this may overstate its role, there is little doubt that self-esteem has a vital impact on individuals' internal (thoughts) and external (behaviors) worlds (31). Self-esteem is relatively stable, and represents an individual's overall feelings of self-competence (32). According to the lifelong concept of self-esteem, socioeconomic status has a long and important influence on the development of individual self-esteem (33). Research has shown that adolescents from higher socioeconomic groups tend to have higher self-esteem (34–36). For example, Yan, Yang (37) found that subjective FSES predicted life satisfaction and that self-esteem mediated this relationship (i.e., adolescents with higher subjective FSES tended to have greater self-esteem, leading to high life satisfaction).

Moreover, self-esteem has been considered to be an internal process and verified as an independent factor that affects various types of interpersonal relationships (38, 39). High self-esteem can help increase the likelihood that adolescents will be accepted by others and buffer the frustration of receiving negative feedback when interacting with others (40–42). It allows people to adjust their reaction to others according to the degree to which they are accepted or rejected by others, and motivates them to respond to others in appropriate ways (41). In general, individuals with high self-esteem exhibit more relationship-strengthening behaviors, while individuals with low self-esteem are more sensitive to rejection, tend to withdraw and reduce interpersonal intimacy after interpersonal conflict, and exhibit more relationship-damaging behaviors (43). However, although research has demonstrated that subjective FSES can affect self-esteem, and self-esteem can be an important predictor of the quality of peer relationships, no study to date has directly explored whether self-esteem plays a mediating effect between subjective FSES and peer relationships. Therefore, one purpose of our study was to explore self-esteem's effect in this association.

In addition to individual psychological characteristics, such as self-esteem, cognitive evaluations of one's external environment, such as perceived stress (29), may also underlie the influence of subjective FSES on peer relationships. Perceived stress is defined as the degree to which people feel that their lives are uncontrollable, unpredictable, or unbearable, and is a cognitive assessment of the severity of the stressor and one's own ability (44, 45); thus, it is a measure of how much stress people have in their lives (46). In some research, assessments of perceived stress has been considered more reliable than assessments of stressful life events (47).

Studies have shown that subjective FSES was significantly associated with perceived stress (46, 48), and adolescents with lower subjective FSES tended to have higher perceived stress. Further, perceived stress was also an important predictor of the quality of interpersonal relationships (41), people with higher perceived stress had lower quality relationships. Previous research conducted in the United States has shown that lower FSES led to more stress and, in turn, lower peer acceptance in children (49). However, Dishion's study (49) only investigated boys and was correlational, while we focused on both genders and added a mediating effect analysis for a more complete understanding.

In summary, some previous studies demonstrated an association between FSES and peer relationships, but few were focused on subjective FSES, and the mechanism of this relationship is still unclear. Thus, to improve our understanding of peer relationships and provide theoretical support for adolescent mental health development, it is important to confirm the potential mechanism.

Therefore, this study explored the association between subjective FSES and peer relationships, and the independent mediating or moderating effect roles of self-esteem and perceived stress. Additionally, some studies have indicated that perceived stress affected adolescents' self-esteem (50, 51). For example, after experiencing high levels of stress, individuals with low self-esteem showed more negative emotions and maladaptive behaviors than individuals with high self-esteem (52). Self-esteem will be threatened when we feel the stressor is uncontrollable or disturbing (29). In other words, high levels of perceived stress can lead to low levels of self-esteem. Therefore, we also investigated the serial mediating effect of perceived stress on self-esteem. The serial mediating effect refers to the existence of multiple mediating variables in the mediating model that form a chain (53); that is, there is mutual influence among the mediating variables. Referring to previous studies, we believed that perceived stress would have an impact on self-esteem.

Based on previous studies, the following hypotheses were proposed:

Hypothesis (1): Lower subjective FSES would be associated with poorer perceived peer relationships.
Hypothesis (2): Perceived stress would mediate the association between subjective FSES and perceived peer relationships.
Hypothesis (3): Self-esteem would mediate the association between subjective FSES and perceived peer relationships.
Hypothesis (4): Subjective FSES would predict perceived peer relationships by the serial mediating effect of perceived stress and self-esteem.

## Materials and Methods

### Participants

A total of 1,790 tenth-grade students from three high schools in Chengdu completed a self-report questionnaire. In consideration of the differences in school conditions, we classified the three high schools according to the education system as national, provincial, and municipal demonstration schools. In total, 737 (24.4%) questionnaires were excluded due to incomplete data, resulting in a valid sample size of 1,353 (75.6%). The participants' ages ranged from 15 to 19 years (*M* = 16.52, *SD* = 0.57), and the sample comprised 623 boys (46.0%) and 730 girls (54.0%).

### Procedures

The students were asked to provide general information on themselves and complete the following questionnaires: MacArthur Scale of Subjective Social Status (54), Rosenberg Self-Esteem Scale (55), Perceived Stress Scale-10 (56), and Peer Relationships subscale (57). It took approximately 30 minutes to complete the questionnaire. In order to ensure confidentiality of participants, a unique ID number was assigned to identify the participant, and all questionnaires were collected then securely stored. All students and their guardians signed a written informed consent form before participation in the study. Students were informed that they could withdraw from the study at any time if they felt uncomfortable responding to the questionnaire items.

### Measures

#### Subjective Family Socioeconomic Status

Developed by Adler, Epel (54), the present study used the MacArthur Scale of Subjective Social Status. It presents a 10-step ladder representing the position of a family; the first step on the ladder represents the bottom, and the tenth represents the top (54). Respondents are asked to choose their location on the ladder according to their family income and occupation. Each step is scored, and the higher the score, the higher the subjective FSES. The scale has shown good reliability [test-retest reliability = 0.76 (58)] and has been used in studies conducted with Chinese adolescents (37, 59, 60). In this study, the Cronbach's alpha coefficient was 0.79.

#### Self-Esteem

Self-esteem was measured using the Rosenberg Self-Esteem Scale (55). This 10-item scale is scored using a four-point Likert scale, ranging from 1 = totally inconsistent to 4 = completely consistent. Items 1, 2, 4, 6, 7, and 8 are positively scored, while items 3, 5, 9, and 10 are reverse scored (61). Scores can range from 10 to 40, with higher scores indicating higher self-esteem. The Cronbach's alpha coefficient in our study was 0.82, and the scale has demonstrated good reliability and validity in prior studies (62, 63).

#### Perceived Stress

The Perceived Stress Scale-10 (PSS-10) was used to measure the degree to which the participants appraised events during the past month as stressful (56). Items are rated using a five-point Likert scale (1 = never, 2 = rarely, 3 = sometimes, 4 = often, and 5 = always). Out of 10 items in total, six are considered negative (items 1, 2, 3, 6, 9, and 10) and assess the respondent's level of distress, while the other four are positive (items 4, 5, 7, and 8) and reflect the respondent's perceived ability to cope with stressors (56). When calculating the total score for the PSS-10, positive items are reverse scored. The total score of this scale ranges from 10 to 50, and the higher the score, the higher the degree of perceived stress. In this study, the Cronbach's alpha coefficient was 0.79, and the scale has shown good reliability and validity in prior studies (64–66).

#### Peer Relationships

We used the Peer Relationship subscale (18 items) of the Self-Description Questionnaire (SDQ) developed by Marsh, Smith (57). The subscale includes two factors to assess perceived relationships with both opposite- and same-gender peers. Items are responded to using a six-point Likert scale ranging from 1 = totally inconsistent to 6 = completely consistent. Scores can range from 18 to 108, and the higher the score, the better the relationship with peers. In this study, the Cronbach's alpha coefficient was 0.86.

#### Control Variables

Some previous studies have suggested that gender and age may influence peer relationships, so we controlled for gender and age in the data analysis (41). In addition, in order to control for the effect of objective FSES, we collected data on the level of education of the parents as an objective indicator. Parental Education has been reported to be strongly associated with self-esteem (37), perceived stress, and peer relationships. In this study, level of education was defined as the years of education.

### Statistical Analysis

Data analysis was performed using SPSS version 22.0. First, this study utilized self-report measurement scales. Although each scale has demonstrated good reliability and validity, the use of common methods could effect the results. Therefore, we addressed the issue of common method bias by conducting a Harman's one-factor test (37), and used exploratory factor analysis (EFA) to validate common method deviations (51). If the rate of explained variance for the first factor is <40%, there is no common method bias (67).

Next, descriptive statistics were used to describe the demographic data, and Spearman correlation analysis was used to examine the relationships between variables. The SPSS Marco PROCESS (models 1, 4, and 6) was used to analyze single and serial multiple mediating or moderating effects after all variables were standardized (53). It was also used to compare the strength of the mediating effects of perceived stress and self-esteem. This method utilizes bias-corrected bootstrapped 95% confidence intervals (CI) based on 5,000 iterations to calculate the mediating effect (68). If the 95% CI does not include 0 and the level of *p* < 0.05, the results are considered significant (69). All analyses were controlled for age and gender.

## Results

### Common Method Bias

The results of the common method bias analysis revealed the rate of explained variance was 22.19%, which was less than the critical value of 40%. This indicated that there was no serious common method bias in this study's data (67).

### Descriptive Statistics and Correlations

The descriptive statistics and bivariate correlations for the four key variables are reported in Table 1. As expected, subjective FSES, self-esteem, perceived stress, and peer relationships were all significantly correlated. Subjective FSES positively correlated with self-esteem (*r* = 0.208, *p* < 0.001). Perceived stress negatively correlated with self-esteem (*r* = −0.493, *p* < 0.001) and subjective FSES (*r* = −0.141, *p* < 0.001). Peer relationships positively correlated with subjective FSES (*r* = 0.232, *p* < 0.001) and self-esteem (*r* = 0.322, *p* < 0.001), and negatively correlated with perceived stress (*r* = −0.267, *p* < 0.001). Objective FSES significantly positively correlated with subjective FSES (*r* = 0.342, *p* < 0.001), self-esteem (*r* = 0.110, *p* < 0.001), and peer relationships (*r* = 0.096, *p* < 0.001), but there was no significant correlation with perceived stress (*r* = −0.043, *p* > 0.05).

**Table 1:** Means, standard deviations, and correlations.

**Variable**	***M***	***SD***	**1**	**2**	**3**	**4**
1. Subjective FSES	5.71	1.469	—			
2. Objective FSES	22.14	5.028	0.342^***^	—		
3. Perceived stress	29.32	5.092	−0.141^***^	−0.043	—	
4. Self-esteem	28.09	3.878	0.208^***^	0.110^***^	−0.493^***^	—
5. Peer relationships	81.91	11.55	0.232^***^	−0.096^***^	−0.267^***^	0.322^***^

*N = 1,353. FSES, family socioeconomic status*.

*** *p < 0.001*.

### Single Mediation Analysis

To test the hypothesis, we conducted a single mediation analysis of the effects of self-esteem and perceived stress in the association between subjective FSES and peer relationships. As hypothesized, perceived stress had a significant mediating effect [*R*^2^ = 0.110, indirect effect = 0.034, 95% CI = (0.020, 0.051), *p* < 0.001], as did self-esteem [*R*^2^ = 0.133, indirect effect = 0.058, 95% CI = (0.040, 0.078), *p* < 0.001].

### Multiple Mediation Analysis

**Figure 1:**
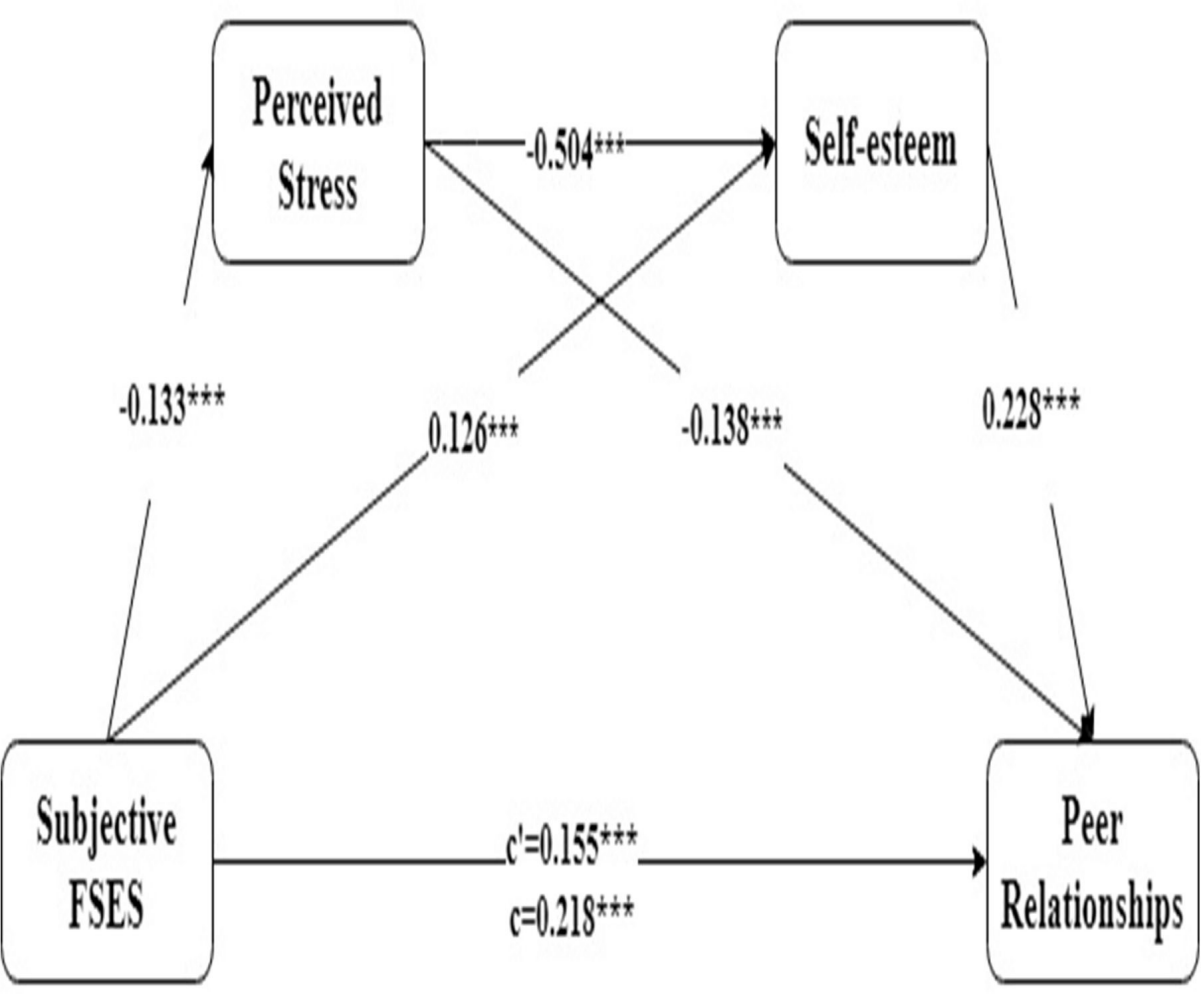
Multiple mediation model of perceived stress and self-esteem in the association between subjective FSES and peer relationships (*N* = 1,353) ***p < 0.001.

The results of the multiple mediation analysis are presented in Figure 1. First, the standardized regression coefficient (β) was significant in each path (*p*s < 0.001). The total effect of subjective FSES on peer relationships was significant (*R*^2^ = 0.147, β = 0.218, *p* < 0.001). When controlling for the mediating variables, the direct effect remained significant (β = 0.155, *p* < 0.001).

Next, as shown in Table 2, the results analyzing subjective FSES → perceived stress → peer relationships (Ind 1: indirect effect = 0.018, 95% CI = (0.009, 0.033)], and FSES → self-esteem → peer relationships [Ind 2: indirect effect = 0.029, 95% CI = (0.017, 0.044)] indicated the bootstrap 95% CIs did not contain 0; therefore, the independent mediating effects of perceived stress and self-esteem were significant. Furthermore, a path from subjective FSES to peer relationships through perceived stress and self-esteem (Ind 3) remained significant [indirect effect = 0.015, 95% CI = (0.009, 0.024)], indicating that the link between subjective FSES and peer relationships could be mediated by the serial path of perceived stress and self-esteem.

**Table 2:** Standardized indirect effects and 95% confidence intervals.

**Model pathway**	**Effect**	**Boot SE**	**Boot LLCI**	**Boot ULCI**	**Relative mediation effect**
Total	0.062	0.010	0.044	0.084	28.44%
Ind 1	0.018	0.006	0.009	0.033	8.26%
Ind 2	0.029	0.007	0.017	0.044	13.30%
Ind 3	0.015	0.004	0.009	0.024	6.88%

*Boot, bootstrap; SE, standard error; LLCI, lower limit confidence interval; ULCI, upper limit confidence interval; Ind 1, subjective family socioeconomic status → perceived stress → peer relationships; Ind 2, subjective family socioeconomic status → self-esteem → peer relationships; Ind 3, subjective family socioeconomic status → perceived stress → self-esteem → peer relationships*.

Finally, the mediating effects of perceived stress and self-esteem were compared using contrasts tests (70). We found that the 95% CI interval of the mediating effect of perceived stress minus the mediating effect of self-esteem contained zero [indirect effect = −0.0105, 95% CI = (−0.031, 0.010)], indicating that there were no significant differences between the mediating effects of perceived stress and self-esteem. In other words, perceived stress and self-esteem were shown to play equally important roles in the association between subjective FSES and peer relationships.

### Supplementary Analyses

We conducted an exploratory moderating analyses to examine the moderated effect of perceived stress and self-esteem by using Hayes' PROCESS model 1. The results showed that the moderating effects of perceived stress were not significant [*R*^2^ = 0.0001, 95% CI = (−0.040, 0.064), *p* > 0.05]. The moderating effect of self-esteem was also not significant [*R*^2^ = 0.0003, 95% CI = (−0.030, 0.065), *p* > 0.05]. These results indicated that perceived stress and self-esteem had no moderating effect in the link of subjective FSES and peer relationships.

In addition, we also tested whether the results were influenced by parent's level of education through multiple mediation analysis. The results revealed that the total effect of subjective FSES on peer relationships was significant (*R*^2^ = 0.147, β = 0.210, *p* < 0.001). When controlling for the mediating variables, the direct effect remained significant (β = 0.152, *p* < 0.001). There was no significant change in each path (see Supplementary Table 1), and the parent's level of education did not play a significant role in the model [*p* > 0.05, and 95% CI = (−0.142, 0.063)] includes 0.

## Discussion

We explored the mediating effects of perceived stress and self-esteem in the association between adolescents' subjective FSES and peer relationships. As expected, the results of the correlation analysis showed that subjective FSES was significantly associated with peer relationships, and self-esteem and perceived stress not only had independent mediating effects on that association but also produced a serial mediating effect. These results indicate that subjective FSES is an important influencing factor in peer relationships, and we hope that by exploring the underlying mechanism, the negative influence of subjective FSES on peer relationships can be reduced.

First, we found a strong positive correlation between subjective FSES and peer relationships, which is in line with previous studies. For example, adolescents from families in the lowest income quintile of in-school families rejected their classmates more than their more advantaged peers (8), while adolescents with higher FSES were more likely to establish positive peer relationships (71). The reason for this phenomenon may be that adolescents' economic status mainly depends on their parents. If their families lack financial resources, adolescents are often unable to participate in group activities and are more likely to experience isolation in school (32). This suggests that we need to focus on the financial resources of adolescents from low-income families. At the same time, schools are encouraged to set up corresponding elective courses in psychology to help students understand the importance of peer relationships, learn the skills and methods of getting along with one another, and adjust students' inaccurate perceptions and comparisons of families based on SES. In addition, schools can provide opportunities for adolescents to participate in group activities by consciously organizing activities that cost less to students, so as to increase the chances of healthy communication among diverse students.

Next, we found that perceived stress played a mediating role in the association between subjective FSES and peer relationships. This is consistent with a previous study showing that lower SES led to higher levels of perceived stress (48). Further, people tend to build good relationships in low-stress environments (72) and maintain poor relationships in high-stress environments (73). Adolescents with higher FSES may face less adversity, receive more support, and be able to afford more options for coping with any problems they encounter (74), which may make them more active in peer relationships. Thus, our study extends previous findings from the United States (49), verifying the mediating role of perceived stress in the link between subjective FSES and peer relationships, and by demonstrating that the role of perceived stress is not limited to boys.

Self-esteem was also found to play a mediating role in the association between subjective FSES and peer relationships. Some studies seem to support our results, as adolescents' subjective FSES has been found to be significantly correlated with self-esteem (35, 75). Moreover, a previous study found that self-esteem was a sociometric that is involved in the maintenance of interpersonal relationships (76), and there is a wealth of research demonstrating the correlation between self-esteem and interpersonal relationships (77, 78). The correlation held for all three interpersonal relationships, including parent-child, teacher-student, and peer relationships (41). Thus, by exploring the mediating role of self-esteem, our study may provide support for future research on the association between subjective FSES and peer relationships.

In addition, we found no significant difference in the mediating effects of self-esteem and perceived stress in the association between subjective FSES and peer relationships, suggesting that they play equally important mediating roles. Adolescence is a time when individuals undergo prominent psychological and physical changes, and adolescents' sense of self-worth and self-awareness continue to increase (79, 80), similar to their awareness of their external environment. Adolescents are in a transitional and weight-bearing period between childhood and adulthood (81, 82). During this period, adolescents start to become more involved in social situations outside the home, which may make them perceive more pressure from their families, school, and social environment (83). Additionally, as they develop a sense of self-consciousness, they focus more on individual differences, thus affecting their self-esteem. This may be why, for adolescents, self-esteem and perceived stress play equally important roles in the association between subjective FSES and peer relationships.

Moreover, we found that the serial mediating effect of perceived stress on self-esteem was significant in the association between subjective FSES and peer relationships, which is consistent with previous research. For example, studies have reported that individuals with higher subjective FSES tended to experience lower stress levels (84), individuals with lower perceived stress had higher self-esteem (50), and self-esteem may affect peer relationships (68). Accordingly, subjective FSES could influence peer relationships through the serial mediation of perceived stress and self-esteem. That is, beyond the independent influences of perceived stress and self-esteem, these two variables have a common influence on this association. In addition, exploration of this serial mediating effect enriched knowledge on the pathways to perceived stress and self-esteem in the association between subjective FSES and peer relationships, and also suggests that we should pay attention to this association in other social psychological mechanisms.

Several limitations of our study should be addressed. First, we used a cross-sectional design, from which no causal inferences can be made. In the future, longitudinal research should be conducted to address this issue. Secondly, we found that the indirect effects of self-esteem and perceived stress were not high. Future research should include other more relevant variables in order to improve our understanding of the mechanism of action in the relationship between subjective FSES and peer relationships. Finally, the sample was from a single cultural background [Chengdu, China; (85)] with a narrow age range; therefore, the generalizability of the results is limited (37).

Although the current study has some limitations, it also has important practical significance. First, we provided a new theoretical framework to explore the mediating effect of perceived stress and self-esteem in the association between subjective FSES and peer relationships among Chinese adolescents. We found that the effect of subjective FSES on peer relationships was not only through individual psychological characteristics (i.e., self-esteem) but also through individual cognitive evaluations (i.e., perceived stress). In addition, the serial mediated path of perceived stress and self-esteem also provides a new direction for future research. Adolescence is an important period of psychological development, and we can improve adolescents' self-esteem and reduce their stress through training interventions and preventive measures, to help them improve their peer relationships and reduce the adverse impact of low subjective FSES.

## Data Availability Statement

The raw data supporting the conclusions of this article will be made available by the authors, without undue reservation.

## Ethics Statement

The studies involving human participants were reviewed and approved by the Medical Ethics Committee of Dali University. Written informed consent to participate in this study was provided by the participants' legal guardian/next of kin.

## Author Contributions

XB and LJ: conceptualization, statistical analysis, and writing the original draft. QZ: revised the manuscript. TW, XZ, and YL: conducted literature searches. SW, LZ, and YL: date collection and writing guidance. YZ and JD: conceptualization, investigation, methodology, and data curation. All authors contributed to the article and approved the submitted version.

## Conflict of Interest

The authors declare that the research was conducted in the absence of any commercial or financial relationships that could be construed as a potential conflict of interest.

## References

[1] Wen M Lin D. Child development in rural China: children left behind by their migrant parents and children of nonmigrant families. Child Dev. (2012) 83:120–36. 10.1111/j.1467-8624.2011.01698.x 22181046

[2] Harris JR. Where is the childs environment - a group socialization theory of development. Psychol Rev. (1995) 102:458–89. 10.1037/0033-295X.102.3.458

[3] Brown BB Larson J. Peer Relationships in Adolescence. John Wiley & Sons (2009). 10.1002/9780470479193

[4] Chen Y Wang L Zhao J. Peer relationship profiles in rural Chinese adolescents: longitudinal relations with subjective well-being. J Health Psychol. (2019). 10.1177/1359105319888278. [Epub ahead of print]. 31749384

[5] Jiao C Wang T Liu J Wu H Cui F Peng X. Using exponential random graph models to analyze the character of peer relationship networks and their effects on the subjective well-being of adolescents. Front Psychol. (2017) 8:583. 10.3389/fpsyg.2017.00583 28450845 PMC5389982

[6] Ladd GW Burgess KB. Charting the relationship trajectories of aggressive, withdrawn, and aggressive/withdrawn children during early grade school. Child Dev. (1999) 70:910–29. 10.1111/1467-8624.00066 10446726

[7] Chen SH ZK. Z. The influence of peer relationship on adolescents mental health. Hunan Norm U J Edu Sci. (2007) 6:76–9.

[8] Hjalmarsson S. Poor kids? economic resources and adverse peer relations in a nationally representative sample of Swedish adolescents. J Youth Adolesc. (2018) 47:88–104. 10.1007/s10964-017-0747-8 28929271 PMC5750326

[9] Novak M AhIgren C Hammarstrom A. Inequalities in smoking: influence of social chain of risks from adolescence to young adulthood: a prospective population-based cohort study. Int J Behav Med. (2007) 14:181–7. 10.1007/BF03000190 18062061

[10] Moor I Rathmann K Lenzi M Pförtner TK Nagelhout GE de Looze M. Socioeconomic inequalities in adolescent smoking across 35 countries: a multilevel analysis of the role of family, school and peers. European Journal of Public Health. (2016) 25:457–63. 10.1093/eurpub/cku244 25713016

[11] Elliott R Leonard C. Peer pressure and poverty: exploring fashion brands and consumption symbolism among children of the ‘British poor'. J Consum Behav. (2004) 3:347–59. 10.1002/cb.147

[12] Chen E Paterson LQ. Neighborhood, family,and subjective socioeconomic status: how do they relate to adolescent health? Health Psychol. (2006) 25:704–14. 10.1037/0278-6133.25.6.704 17100499

[13] Operario D Adler NE Williams DR. Subjective social status: reliability and predictive utility for global health. Psychol Health. (2004) 19:237–46. 10.1080/08870440310001638098

[14] Singh-Manoux A Marmot MG Adler NE. Does subjective social status predict health and change in health status better than objective status? Psychosom Med. (2005) 67:855–61. 10.1097/01.psy.0000188434.52941.a0 16314589

[15] Zell E Strickhouser JE Krizan Z. Subjective social status and health: a meta-analysis of community and society ladders. Health Psychol. (2018) 37:979–87. 10.1037/hea0000667 30234357

[16] Lerner RM DR C. Contemporary developmental theory and adolescence: developmental systems and applied developmental science. J Adolesc Health. (2002) 31:122–35. 10.1016/S1054-139X(02)00495-0 12470909

[17] Cox MJ Paley B. Families as systems. Ann Rev Psychol. (1997) 48:243–67. 10.1146/annurev.psych.48.1.243 9046561

[18] Bronfenbrenner U. Toward an experimental ecology of human development. Am Psychol. (1979) 32:513–31. 10.1037/0003-066X.32.7.513

[19] Chen F L X. Impact of family function on externalizing problem behavior among rural children left behind: on mediation of self-esteem. J Human Agric Univ. (2016) 17:67–70. 10.13331/j.cnki.jhau(ss).2016.01.011

[20] Harter S. The Construction of the Self. 2nd ed. New York, NY: Guilford Press (2012).

[21] Amato SPR. Economic hardship in the family of origin and children's psychological well-being in adulthood. J Marriage Fam. (2005) 67:141–56. 10.1111/j.0022-2445.2005.00011.x

[22] Kavanaugh SA Neppl TK Melby JN. Economic pressure and depressive symptoms: testing the family stress model from adolescence to adulthood. J Fam Psychol. (2018) 32:957–65. 10.1037/fam0000462 30070569 PMC6205903

[23] Han S. Social capital and perceived stress: the role of social context. J Affect Disord. (2019) 250:186–92. 10.1016/j.jad.2019.03.034 30856496

[24] Masarik AS Conger RD. Stress and child development: a review of the family stress model. Curr Opin Psychol. (2017) 13:85–90. 10.1016/j.copsyc.2016.05.008 28813301

[25] Lucasthompson RG Goldberg WA. Family relationships and children's stress responses. Adv Child Dev Behav. (2011) 40:243–99. 10.1016/B978-0-12-386491-8.00007-4 21887964

[26] Taylor RD Rodriguez AU Seaton EK Dominguez A. Association of financial resources with parenting and adolescent adjustment in african american families. J Adolesc Res. (2004) 19:267–83. 10.1177/0743558403258828

[27] Taylor RD Budescu M Gebre A Hodzic I. Family financial pressure and maternal and adolescent socioemotional adjustment: moderating effects of kin social support in low income African American Families. J Child Fam Stud. (2014) 23:242–54. 10.1007/s10826-012-9688-8

[28] Ponnet K. Financial stress, parent functioning and adolescent problem behavior: an actor–partner interdependence approach to family stress processes in low-, middle-, and high-income families. J Youth Adolesc. (2014) 43:1752–69. 10.1007/s10964-014-0159-y 25053382

[29] Lee JS Joo EJ Choi KS. Perceived stress and self-esteem mediate the effects of work-related stress on depression. Stress Health. (2013) 29:75–81. 10.1002/smi.2428 22610597

[30] Mecca AW Smelser NJ Vasconcellos J. The Social Importance of Self-Esteem. Berkeley, CA: University of California Press (1989).

[31] Cameron JJ Granger S. Does self-esteem have an interpersonal imprint beyond self-reports? a meta-analysis of self-esteem and objective interpersonal indicators. Person Soc Psychol Rev. (2019) 23:73–102. 10.1177/1088868318756532 29482451

[32] Hjalmarsson S Mood C. Do poorer youth have fewer friends? The role of household and child economic resources in adolescent school-class friendships. Children Youth Serv Rev. (2015) 57:201–11. 10.1016/j.childyouth.2015.08.013

[33] Orth U Trzesniewski KH Robins RW. Self-esteem development from young adulthood to old age: a cohort-sequential longitudinal study. J Pers Soc Psychol. (2010) 98:645–58. 10.1037/a0018769 20307135

[34] Haught HM Rose J Geers A Brown JA. Subjective social status and well-being: the role of referent abstraction. J Soc Psychol. (2015) 155:356–69. 10.1080/00224545.2015.1015476 25668216

[35] Hayashida T Higashiyama M Sakuta K Masuya J Ichiki M Kusumi I. Subjective social status via mediation of childhood parenting is associated with adulthood depression in non-clinical adult volunteers. Psychiatry Res. (2019) 274:352–7. 10.1016/j.psychres.2019.02.061 30851598

[36] Twenge JM Campbell WK. Self-esteem and socioeconomic status: A meta-analytic review. Person Soc Psychol Rev. (2002) 6:59–71. 10.1207/S15327957PSPR0601_3

[37] Yan W Yang K Wang Q You X Kong F. Subjective family socioeconomic status and life satisfaction in Chinese adolescents: the mediating role of self-esteem and social support. Youth Society. (2020) 11:1–19. 10.1177/0044118X20941344

[38] Andreopoulou A Houston DM. The impact of collective self-esteem on intergroup evaluation: self-protection and self-enhancement. Curr Res Soc Psychol. (2002) 7:243–56.

[39] Vohs KD Heatherton TF. The effects of self-esteem and ego threat on interpersonal appraisals of men and women: a naturalistic study. Pers Soc Psychol Bull. (2003) 29:1407–20. 10.1177/0146167203255767 15189578

[40] McFarland C Buehler R. Collective self-esteem as a moderator of the frog-pondeffect in reactions to performance feedback. J Person Soc Psychol. (1995) 68:1055–70. 10.1037/0022-3514.68.6.1055

[41] Bi Y Ma L Yuan F Zhang B. Self-Esteem, perceived stress, and gender during adolescence: interactive links to different types of interpersonal relationships. J Psychol. (2016) 150:36–57. 10.1080/00223980.2014.996512 25584816

[42] Brown JD. High self-esteem buffers negative feedback: once more with feeling. Cogn Emot. (2010) 24:1389–404. 10.1080/02699930903504405

[43] Orth U Robins RW Widaman KF. Life-span development of self-esteem and its effects on important life outcomes. J Pers Soc Psychol. (2012) 102:1271–88. 10.1037/a0025558 21942279

[44] Luethi M Meier B Sandi C. Stress effects on working memory, explicit memory, and implicit memory for neutral and emotional stimuli in healthy men. Front Behav Neurosci. (2008) 2:5. 10.3389/neuro.08.005.2008 19169362 PMC2628592

[45] Jimenez TI Estevez E Velilla CM Martin-Albo J Martinez ML. Family communication and verbal child-to-parent violence among adolescents: the mediating role of perceived stress. Int J Environ Res Public Health. (2019) 16:4538. 10.3390/ijerph16224538 31744062 PMC6888577

[46] Feizi A Aliyari R Roohafza H. Association of perceived stress with stressful life events, lifestyle and sociodemographic factors: a large-scale community-based study using logistic quantile regression. Comput Math Methods Med. (2012) 2012:151865. 10.1155/2012/151865 23091560 PMC3471433

[47] Cohen S. Contrasting the hassles scale and the perceived stress scale: who's really measuring appraised stress? Am Psychol. (1986) 41:716–8. 10.1037/0003-066X.41.6.716

[48] Ursache A Noble KG Blair C. Socioeconomic status, subjective social status, and perceived stress: associations with stress physiology and executive functioning. Behav Med. (2015) 41:145–54. 10.1080/08964289.2015.1024604 26332932 PMC4722863

[49] Dishion TJ. The family ecology of boys' peer relations in middle childhood. Child Dev. (1990) 61:874–92. 10.2307/1130971 2364761

[50] Wilson M Thayer Z. Impact of acculturation on depression, perceived stress and self-esteem in young Middle Eastern American adults. Ann Hum Biol. (2018) 45:346–53. 10.1080/03014460.2018.1484160 30200786

[51] Guo L Tian L Scott Huebner E. Family dysfunction and anxiety in adolescents: a moderated mediation model of self-esteem and perceived school stress. J Sch Psychol. (2018) 69:16–27. 10.1016/j.jsp.2018.04.002 30558751

[52] Moksnes UK Moljord IEO Espnes GA Byrne DG. The association between stress and emotional states in adolescents: the role of gender and self-esteem. Person Ind Differ. (2010) 49:430–5. 10.1016/j.paid.2010.04.012

[53] Hayes AF. Introduction to mediation, moderation, and conditional process analysis: a regression-based approach. J Educ Meas. (2013) 51:335–7. 10.1111/jedm.12050

[54] Adler NE Epel ES Castellazzo G Ickovics JR. Relationship of subjective and objective social status with psychological and physiological functioning: preliminary data in healthy white women. Health Psychol. (2000) 19:586–92. 10.1037/0278-6133.19.6.586 11129362

[55] Rosenberg M. Society and the adolescent self-image. Princeton. (1965) 3:1780–90. 10.1515/9781400876136

[56] Sun Y Gao L. The perceived stress scale-10 (PSS-10) is reliable and has construct validity in Chinese patients with systemic lupus erythematosus. Lupus. (2019) 28:149–55. 10.1177/0961203318815595 30518288

[57] Marsh HW Smith ID Barnes J. Multidimensional self-concepts: relationships with inferred self-concepts and academic achievement. Aust J Psychol. (1984) 36:367–86. 10.1080/00049538408255318

[58] Hu M Wang M Cai L Zhu X. Development of subjective socioeconomic status scale for Chinese adolescents. Chin J Clin Psychol. (2012) 20:155–61. 10.16128/j.cnki.1005-3611.2012.02.026

[59] Chen Y Yao S Xia L. Validity and reliability of the Chinese version of the Subjective Socioeconomic Status Scale in a general adult population. Chin J Mental Health. (2014) 28:869–74.

[60] Wang S Zhao Y Li J Lai H Qiu C Pan N. Neurostructural correlates of hope: dspositional hope mediates the impact of the SMA gray matter volume on subjective well-being in late adolescence. Social Cogn Affect Neurosci. (2020) 15:395–404. 10.1093/scan/nsaa046 32378710 PMC7308655

[61] Greenberger E Chuansheng C Dmitrieva J. Item-wording and the dimensionality of the Rosenberg Self-esteem Scale: do they matter? Person Ind Differ. (2003) 35:1241–54. 10.1016/S0191-8869(02)00331-8

[62] Kong F Ding K Zhao J. The relationships among gratitude, self-esteem, social support and life satisfaction among undergraduate students. J Happ Stud. (2015) 16:477–89. 10.1007/s10902-014-9519-2

[63] Wang K Kong F. Linking trait mindfulness to life satisfaction in adolescents: the mediating role of resilience and self-esteem. Child Indic Res. (2020) 13:321–35. 10.1007/s12187-019-09698-4

[64] Lu W Bian Q Wang W Wu X Wang Z Zhao M. Chinese version of the perceived stress scale-10: a psychometric study in Chinese university students. PLoS ONE. (2017) 12:e0189543. 10.1371/journal.pone.0189543 29252989 PMC5734731

[65] Wang Z Chen J Boyd JE. Psychometric properties of the Chinese version of the perceived stress scale in policewomen. PLoS ONE. (2011) 6:e28610. 10.1371/journal.pone.0028610 22164311 PMC3229602

[66] Liu X Zhao Y Li J Dai J Wang X Wang S. Factor structure of the 10-item perceived stress scale and measurement invariance across genders among Chinese adolescents. Front Psychol. (2020) 11:537. 10.3389/fpsyg.2020.00537 32328009 PMC7160845

[67] Podsakoff PM MacKenzie SB Lee JY Podsakoff NP. Common method biases in behavioral research: a critical review of the literature and recommended remedies. J Appl Psychol. (2003) 88:879–903. 10.1037/0021-9010.88.5.879 14516251

[68] Blairy S Linotte S Souery D Papadimitriou G Dikeos D Lerer B. Social adjustment and self-esteem of bipolar patients: amulticentric study. J Affect Disord. (2004) 79:97–103. 10.1016/S0165-0327(02)00347-6 15023484

[69] Preacher KJ Hayes AF. Asymptotic and resampling strategies for assessing and comparing indirect effects in multiple mediator models. Behav Res Methods. (2008) 40:879–91. 10.3758/BRM.40.3.879 18697684

[70] Zhao J Song F Chen Q Li M Wang Y Kong F. Linking shyness to loneliness in Chinese adolescents: the mediating role of core self-evaluation and social support. Person Ind Differ. (2018) 125:140–4. 10.1016/j.paid.2018.01.007

[71] Ye Z Wen M Wang W Lin D. Subjective family socio-economic status, school social capital, and positive youth development among young adolescents in China: a multiple mediation model. Int J Psychol. (2020) 55:173–81. 10.1002/ijop.12583 31066032

[72] Pau AK Croucher R. Emotional intelligence and perceived stress in dental undergraduates. J Dent Educ. (2003) 67:1023–8. 10.1002/j.0022-0337.2003.67.9.tb03685.x 14518841

[73] Forushani NZ Besharat MA. Relation between emotional intelligence and perceived stress among female students. Proc Soc Behav Sci. (2011) 30:1109–12. 10.1016/j.sbspro.2011.10.216

[74] Baum A Garofalo JP Yali AM. Socioeconomic status and chronic stress. Does stress account for SES effects on health? Ann N Y Acad Sci. (1999) 896:131–44. 10.1111/j.1749-6632.1999.tb08111.x 10681894

[75] Cook WK Mulia N Li L. Subjective social status and financial hardship: associations of alternative indicators of socioeconomic status with problem drinking in Asian Americans and Latinos. Substance Use Misuse. (2020) 55:1246–56. 10.1080/10826084.2020.1732423 32133915 PMC7837702

[76] Leary MR. Responses to social exclusion: social anxiety, jealousy, loneliness, depression, and low self-esteem. J Soc Clin Psychol. (1990) 9:221–9. 10.1521/jscp.1990.9.2.221

[77] Barker V. Older adolescents' motivations for social network site use: the influence of gender,group identity, and collective self-esteem. Cyber Psychol Behav. (2009) 12:209–13. 10.1089/cpb.2008.0228 19250021

[78] Mao HY Chen CY Hsieh TH. The relationship between bureaucracy and workplace friendship. Soc Behav Person. (2009) 37:255–66. 10.2224/sbp.2009.37.2.255

[79] Chu L Powers PA. Synchrony in adolescence. Adolescence. (1995) 30:453–61. 7676879

[80] Noom MJ Dekovic M Meeus WHJ. Conceptual analysis and measurement of adolescent autonomy. J Youth Adolesc. (2001) 30:577–95. 10.1023/A:1010400721676

[81] Sawyer SM Azzopardi PS Wickremarathne D Patton GC. The age of adolescence. Lancet Child Adolesc Health. (2018) 2:223–8. 10.1016/S2352-4642(18)30022-1 30169257

[82] Lenjalley A Radjack R Ludot M Touhami F Moro MR. Adolescent vulnerabilities and radicalisation. Soins. (2017) 62:38–42. 10.1016/j.soin.2017.08.009 29031381

[83] Pinto AA Claumann GS Medeiros P Barbosa R Nahas MV Pelegrini A. Association between perceived stress in adolescence, body weight and romantic relationships. Rev Paul Pediatr. (2017) 35:411–28. 10.1590/1984-0462/;2017;35;4;00012 28977133 PMC5737262

[84] Senn TE Walsh JL Carey MP. The mediating roles of perceived stress and health behaviors in the relation between objective, subjective, and neighborhood socioeconomic status and perceived health. Ann Behav Med. (2014) 48:215–24. 10.1007/s12160-014-9591-1 24648016 PMC4156915

[85] Li J Zhao Y Kong F Du S Yang S Wnag S. Psychometric assessment of the short grit scale among Chinese adolescents. J Psychoeduc Assess. (2018) 36:291–6. 10.1177/0734282916674858

