# Supplementary material for: Subjective Family Socioeconomic Status and Peer Relationships: Mediating Roles of Self-Esteem and Perceived Stress
Source: Front Psychiatry. 2021 Mar 24;12:634976. doi: 10.3389/fpsyt.2021.634976 (PMC8024469; doi:10.3389/fpsyt.2021.634976)
Supplement: Supplementary file 1 [file Table_1.pdf]

Supplementary analyses

We conducted an exploratory moderating analyses to examine the moderated effect of perceived stress and self-esteem by using Hayes' PROCESS model 1. The results showed that the moderating effects of perceived stress were not significant (R^2 = 0.0001, 95% CI = [-0.040, 0.064], p > 0.05). The moderating effect of self-esteem was also not significant (R^2 = 0.0003, 95% CI = [-0.030, 0.065], p > 0.05). These results indicated that perceived stress and self-esteem had no moderating effect in the link of subjective FSES and peer relationships.

In addition, we also tested whether the results were influenced by parent's level of education through multiple mediation analysis. The results revealed that the total effect of subjective FSES on peer relationships was significant (R^2 = 0.147, β = 0.210, $p < 0.001$). When controlling for the mediating variables, the direct effect remained significant (β = 0.152, $p < 0.001$). There was no significant change in each path (see Table S1), and the parent's level of education did not play a significant role in the model ($p > 0.05$, and 95% CI = [-0.142, 0.063]) includes 0.

Table S1. Standardized indirect effects and 95% confidence intervals (Parental Education were controlled)

Model pathway	Effect	Boot *SE*	Boot LLCI	Boot ULCI	Relative mediation effect
Total	0.058	0.010	0.040	0.080	27.62%
Ind 1	0.018	0.006	0.008	0.032	8.57%
Ind 2	0.025	0.007	0.013	0.041	11.90%
Ind 3	0.015	0.004	0.009	0.024	7.14%

Note: FSES = family socioeconomic status; Boot = bootstrap; SE = standard error; LLCI = lower limit confidence interval; ULCI = upper limit confidence interval; Ind 1 = subjective FSES→perceived stress→peer relationships; Ind 2 = subjective FSES→self-esteem→peer relationships; Ind 3 = subjective FSES→perceived stress→self-esteem→peer relationships.
